# Patterns of Complementary and Alternative Medicine Use, Perceived Benefits, and Adverse Effects among Adult Users in Enugu Urban, Southeast Nigeria

**DOI:** 10.1155/2014/239372

**Published:** 2014-04-02

**Authors:** Ijeoma Okoronkwo, Jane-lovena Onyia-pat, Pat Okpala, Mary-Ann Agbo, Afam Ndu

**Affiliations:** ^1^Department of Nursing Sciences, University of Nigeria, Enugu Campus, Enugu, Nigeria; ^2^School of Post Basic Nursing, Federal Neuropsychiatric Hospital, Enugu, Nigeria

## Abstract

The use of complementary and alternative medicine (CAM) is now on the increase. Evidence from studies carried out globally has established that CAM use is very common and varies among populations. This study investigated patterns of CAM use, perceived benefits, and associated harm with CAM use among adults. A cross-sectional study was conducted in three local government areas of Enugu urban, Southeast Nigeria. An interviewer-administered questionnaire was used to collect data from all consenting adult participants aged between 18 and 65 years. Of the 732 participants interviewed, 62.8% were females while 37.2% were males. Majority (84.7%) of the participants had used CAM at one time or another. The most commonly used CAM product was the biological products, followed by spiritual therapy. The major route of administration for CAM products was oral and about 40% of the participants combined CAM with conventional medicine. Majority (78.6%) of CAM users benefited from CAM products after using them while a few complained of adverse reactions. As CAM is gaining widespread acceptance and use, there is need for clinical trial on the benefits and adverse effects associated with the use of CAM to facilitate proof of efficacy and safety of the products.

## 1. Introduction

The National Centre for Complementary and Alternative Medicine (NCCAM) [1] defines CAM as “a group of various medical and health care systems, practices, and products that are not presently considered to be an aspect of conventional medicine.” Complementary interventions are healthcare approaches used in conjunction with conventional interventions, whereas alternative medicines are used in place of conventional medicine [2].

Complementary and alternative medicine (CAM) is now in common use globally and varies among populations [3]. Reports on the prevalence of CAM use vary greatly in both developed and developing countries [2–5]. Figures have ranged from 7% to 80% [3–5]. In Nigeria, studies carried out on prevalence of CAM use were among cancer patients and a prevalence rate of 65.0% was recorded at the University of Nigeria Teaching Hospital (UNTH), Enugu, Nigeria [4] and 31.1% among children with chronic health conditions in Lagos Teaching hospital (LUTH) [2]. Forms of CAM therapies mostly consumed include herbal medicine, spiritual healing, relaxation techniques, aloe vera, medicinal tea, and nutritional supplements [4, 6–8]. Many reasons have been given for the use of CAM which include treating or preventing diseases, improving the quality of life, and promoting and maintaining health [3, 6].

Herbal CAM therapies are frequently obtained from traditional herbal medicine practitioners [9, 10]. About 85% of Nigerians are known to use and consult traditional medicine for healthcare, social, and psychological benefits because of poverty and dissatisfaction with conventional medical care [2]. Among numerous herbal medicines in circulation in Nigeria, only about twenty have been registered by the National Agency for Food and Drug Agency and Control (NAFDAC) [11]. Yet many unregistered CAM practitioners adopt aggressive marketing strategies in print and electronic media to market their products which are freely available on the open market.

A good number of CAM users take it concurrently with conventional medicine [4, 12–14]. However, some patients who use herbal products are reluctant to disclose use of CAM to their doctors [4] either due to fear of physician's criticism or because the physician failed to ask [4, 8]. In addition, most users of CAM practice self-medication without guide or supervision from licensed or certified CAM practitioners [3, 4, 12]. A study on the use of complementary and alternative medicine by cancer patients at the University of Nigeria Teaching Hospital, Enugu, Nigeria [4], showed that only 21.2% of the patients studied reported unwanted side effects from CAM treatment. The side effects included full thickness chemical burns following application of herbal product on the skin, slimming down, anorexia, nausea and vomiting, general malaise, and diarrhea.

Many studies have reported potential benefits from the use of CAM which include relief of pain, feeling healthy/good, reduction of swelling, relief of constipation, and wound healing [4, 15, 16]. Although CAM has long been in use, there is little evidence regarding its safety and efficacy [1, 6–8]. Majority of those who use CAM products perceive them to be natural and safe [3, 4] and are unaware of their composition and potential harmful effects. Poor quality control of CAM and the coadministration of CAM and conventional treatment may result in adverse reactions [17, 18]. This poses a threat to the life of the individual in particular and the public in general.

Many studies on CAM in Nigeria have focused on patients with chronic conditions. A better understanding of the pattern of CAM use among the adult population is crucial bearing in mind the increased consumption of CAM products globally. This study therefore investigated patterns of CAM use, perceived benefits, and adverse effects among adult population in Enugu urban, south east Nigeria.

## 2. Methods

### 2.1. Research Design and Study Participants

A cross-sectional descriptive survey was used and the study area was drawn from three local government areas in Enugu urban. Enugu urban is the capital of Enugu state which is made up of Enugu North, South, and East Local Government Areas (L.G.As). Being the capital city it has a large concentration of adults who are engaged in various activities. The population of the study comprised adults between the ages of 18–65 years who were residing in Enugu urban at the time of the study. The upper age limit of 65 years was used as a benchmark age limit for work force retirement which falls within 60–65 years depending on the work sector. This age group between 18 and 65 years was therefore assumed to be capable of taking decisions and actions concerning their health. The current 2006 census records the population of Enugu urban to be 722,644 [19].

The minimum sample size of 1000 was determined using Taro Yamene formula [20]:

(1) $$\begin{array}{c} n=\frac{N}{1+N{\left(e\right)}^{2}}, \end{array}$$

where *n* = sample size, *N* = total population of study, 1 = a constant, and *e* ^2^  (0.05^2^) = level of significance.

This is a statistical technique used to determine sample size for a known population. To select the respondents, a multistage sampling technique was used. The first stage involved stratifying Enugu urban into the existing three L.G.As. The second phase involved the use of random sampling to select two residential areas from each of the L.G.As making a total of six residential areas. The third stage involved the use of simple random sampling to select 30 out of the estimated 259 streets in the residential areas. Systematic random sampling was used in the fourth stage to select 1000 houses from the selected streets. Thus, one adult from each house constituted a sampling unit.

### 2.2. Data Collection

A validated interviewer-administered questionnaire was used to collect data from each participant. The questionnaire was developed from previous studies on CAM use. It was used to obtain sociodemographic data on age, gender, marital status, education, income, and religion. Data were also obtained on the prevalence, forms of CAM products consumed, route of administration, coadministration of CAM with conventional medicine, perceived benefits, and adverse reactions associated with the use of CAM. The questions were both open and closed ended. For participants who were not literate, the questionnaire was interpreted to them in the local dialect. CAM use was considered in this study as the use of it either as a complement or an alternative therapy according to the classification of CAM by the National Institute of Health. Patterns of CAM as used refer to the type of CAM consumed, route of administration, and coadministration of CAM with conventional medicine.

### 2.3. Ethical Considerations

The approval for the study was obtained from the Enugu State Ministry of Health ethical committee. Subsequently, informed consent was completed by each study participant who agreed to participate after the study had been explained in English or local dialect. Each participant was informed that participation was voluntary and assured of anonymity and confidentiality of information. They were all given the option of not participating in the study if they wished so. Data collection lasted for one month.

### 2.4. Data Analysis

Data generated were analyzed descriptively. In addition sociodemographic data were analysed inferentially. CAM usage was tested by comparing sociodemographic data of CAM users with nonusers using chi-square at 0.05 level of significance. All statistical analysis was performed using the statistical package for social sciences (SPSS) version 17.0.

## 3. Results

The number of properly filled copies of questionnaire available for data analysis was 732 giving a return rate of 73.2%. Some copies of the completed questionnaires were mutilated, while others were wrongly filled and were discarded as they could not be used for data analysis. Thus 732 respondents constituted the sample size.

### 3.1. Sociodemographic Characteristics of Respondents

Of the 732 participants, 272 (37.2%) were males and 460 (62.8%) females. Six hundred and twenty (84.7%) participants had used CAM at one time or another while 112 (34.3%) had not. Mean age of participants was 37.6 years (SD = 1.28) (see Table 1). Gender, marital status, education level, and income had statistical significant relationships with the use of CAM. Although a greater number of the age group between 26 and 41 years used CAM more than other age groups, these differences were not statistically significant.

### 3.2. Patterns of CAM Use

Analyses on pattern of CAM use, perceived benefits, and associated harm were confined to CAM users only (*n* = 620).

#### 3.2.1. Type of CAM Used by Respondents

Biological products, 347 (56%), were the most frequently used CAM with honey and herbal preparations being the most frequently used products in this category. This was followed by spiritual therapy, 306 (49.4%). Other forms used include physical therapy (massage), black stone, python fat, and crude oil (see Table 2).

#### 3.2.2. Route of Administration for CAM

The major route of administration for CAM among the respondents was oral, 540 (87.1%). This was followed by respondents who used CAM by reciting or reading it while others massaged CAM products on their body (Table 2).

#### 3.2.3. Co-Use of CAM with Conventional Treatment

Over half, 349 (56.3%), of the respondents used CAM alone for treatment and 271 (43.7%) used conventional medicine alone while 248 (40%) combined CAM and conventional medicine together (Table 2).

### 3.3. Perceived Benefits of CAM Products

About 476 (76.8%) of the adults felt they observed specific benefits after using CAM. These include the use of honey for wound dressing and cough relieving, herbal drugs in the treatment of malaria, typhoid fever, and stomach upset, prayer, and massage (Table 3). Four hundred and fifty-three (73.1%) respondents perceived CAM to be effective because it improved their health status and 16 (2.5%) observed no benefit, while 151 (24.4%) could not tell any difference in the use of CAM products (Table 3).

### 3.4. Perceived Adverse Effect of CAM

Majority of the respondents, 436 (70.3%), did not experience any adverse effect after using CAM products while 184 (29.7%) reported having experienced adverse reactions (Table 4). Specific adverse reactions to CAM products observed were generalized body discomfort from using forever living product, dizziness and body weakness from taking herbs, diarrhea from “Tuja 1000,” and stomach discomfort from honey while another effect mentioned was increase in body weight (Table 4).

## 4. Discussion

This study recorded a high prevalence (84.7%) of CAM use among the adult participants. This rate is higher than (65%) previously reported in the use of CAM by cancer patients in University Teaching hospital (UNTH), Enugu, and (31%) in children with chronic conditions in Lagos University Teaching Hospital (LUTH), Nigeria [2, 4]. This variation could be as a result of the population group studied. Many Nigerians use and consult traditional medicine practitioners for healthcare despite current emphasis on conventional treatment [2].

Males were more inclined to use CAM than females in this study. This finding is not consistent with studies in developed countries where women had higher prevalence of use than men [21, 22]. This greater male gender use may be attributed to the African man's strong belief in traditional medicine and his inability to squeeze out time from his busy schedule to visit conventional hospitals [4]. In addition, hospitals are perceived by consumers to be time-wasting and expensive while CAM is seen as being accessible, affordable, and not time-consuming.

Levels of education and income were associated with the use of CAM. This is supported by other studies where age, socioeconomic status, and level of education have been linked to higher prevalence of CAM use [5]. However, there was no link between age and use of CAM in this study. This finding is similar to reports from other developing countries where age had no relationship with CAM use [3, 4, 7].

CAM products used by respondents in this study were consistent with most frequently used CAM products in literature [3–5]. The biological products (honey, herbal preparation) were the most frequently used CAM products by the respondents in the treatment of diseases. This is supported by findings in the United States where herbal preparations were found to be the most common form of CAM used among the elderly [23]. Spiritual therapy was another form of CAM therapy used by the respondents. This finding is supported by Singh et al. who recorded that herbs and spiritual healing were the two most common forms of CAM used among Indians in South Africa [7]. Religion has always enjoyed high favour with most African communities and this could be responsible for the increased number of people who used prayer/faith healing to ease unfavourable health conditions [4].

Majority of CAM users in this study consumed CAM products orally. This finding agrees with the study conducted in South Iran where 99.1% of pregnant women studied consumed CAM orally [13]. Although, most of the respondents in this study did not use CAM in combination with conventional medicine, about 40% used CAM together with conventional medicine. This finding is similar to other reported studies in developed and developing countries where outpatients used CAM in combination with conventional medicines [4, 12–14]. The use of CAM together with conventional medicine can lead to unanticipated reactions [1]. For instance herbal products such as St. John's wort and ginko, which are available over the counter, have been reported to cause serious clinical interactions when taken in combination with prescribed drugs [24]. Combining St. John's wort with certain antidepressants can lead to a potentially life-threatening increase of serotonin, a brain chemical targeted by antidepressants [3]. Other researchers [25, 26] have equally noted that herbal products such as ginseng, ginkgo, garlic, and* echinacea* when used preoperatively can interact with conventional medicine, by altering electrolytes causing increasing bleeding times, prolonged anaesthesia, stroke, and myocardial infarction. It is therefore necessary to obtain history on herbal medication use from patients prior to surgery to avoid herbal-drug interactions that are likely to cause serious side effects.

Most of the respondents benefited from their CAM treatment. Such benefits include honey (for the relief of cough and for wound dressing) and herbal preparations for the treatment of malaria, typhoid fever, and stomach upset, while others benefited from prayers. These findings are consistent with other studies where patients claimed they benefited from herbal preparations, spiritual healing, and honey [4, 5]. The use of honey to enhance wound healing is an established practice in many notable plastic surgical units globally [7, 27]. The perceived benefits of CAM use could also be due to the fact that the disease process being treated had run its natural course or the perceived conditions were anxiety/emotion/psychologically based.

Although CAM is often perceived as being natural and safe with low or no side effects [5], studies have highlighted the possible side effects of certain CAM therapies like ginkgo, ginseng, and St. John's wort when taken in combination with conventional treatment [1, 3, 7]. In this study only 29.7% of the respondents reported adverse reactions to CAM therapies. Specific adverse reactions to CAM observed were generalized body discomfort, dizziness, general malaise/weakness of the body, stomach upset, and diarrhea. These findings are similar to that reported by Fakeye et al. [28] in their study on “Attitude and use of herbal medicines among pregnant women in Nigeria” where 18% of those taking herbals had some form of unwanted effects such as vomiting and dizziness. With increasing CAM use among the adult population, there is need for regulation by the appropriate authority to ensure evidence of safety, efficacy, and rational use of CAM.

This study has some limitations. It was confined to adults in Enugu urban; therefore findings cannot be generalized to all adults in Enugu state or the whole country. Furthermore the high default rate is a limitation of the study which should be taken care of in future research. A more qualitative study on CAM use is essential to verify the benefits and adverse effects associated with CAM use.

## 5. Conclusion

This study showed a high prevalence of CAM use among adult populations in Enugu urban. Major forms of CAM products consumed by the respondents were biological and spiritual therapy. The major route for CAM consumption was oral. Although majority took CAM singly, about 40% took it concurrently with conventional treatment. Perceived benefit and effectiveness of CAM treatment on their health condition was high; however, few respondents reported adverse reactions. This finding is important because of the risk involved in herbal- drug reaction which may pose a threat to individual health in particular and public health in general. Findings suggest a need for clinical trials to determine the safety and efficacy of CAM products due to increased consumption by the general population.

## Figures and Tables

**Table 1:** Demographic characteristics of CAM users and nonusers *n* = 732.

Parameters	CAM users	Non-CAM users	*P* value*
Sex			0.004*
Male	244 (39.4%)	28 (25%)	
Female	376 (60.6%)	84 (75%)	
Age in years			0.267
18–25	76 (12.3%)	18 (16.0%)	
26–33	189 (30.5%)	29 (25.9%)	
34–41	184 (29.6%)	25 (22.3%)	
42–49	71 (11.5%)	14 (12.5%)	
50–57	40 (6.5%)	10 (8.9%)	
58–65	60 (9.6%)	16 (14.3%)	
Total	**620**	**112**	
Mean age $\left(\overset{-}{x}\right)$	37.4 years	38.7 years	
Marital status			0.030*
Never married	213 (34.7%)	33 (29.5%)	
Married	370 (59.7%)	60 (83.6%)	
Divorced/separated	9 (1.4)	3 (2.7%)	
Widow/widower	28 (4.5%)	12 (0.7%)	
Level of education			0.056*
No formal	6 (10%)	5 (4.4%)	
Primary	25 (4.0%)	7 (6.3%)	
Postprimary	226 (36.5%)	46 (41.1%)	
Tertiary	363 (58.5%)	54 (48.2%)	
Level of income			0.014*
Low income (≤#50,000/month)	479 (77.2%)	63 (56.2%)	
Middle income (#50,000–100,000/month)	111 (18.9%)	30 (26.8%)	
High income (≥#100,000)	24 (3.9%)	6 (5.4%)	
Religion			
Christianity	616 (99.3%)	111 (99.1)	
Moslem	1 (0.2%)	1 (0.9%)	
Traditional religion	3 (0.5%)	0	

*Significance at 0.05 level.

**Table 2:** Pattern of CAM.

Item	*f*	%
Forms of CAM used		
Biological products	347	56
Spiritual therapy	306	49.4
Physical therapy	137	22.1
Alternative medicine	45	7.3
Others (urine therapy, python fat, and black stone)	140	26.6
Route of CAM consumption		
Oral	540	87.1
Recitation/reading	306	49.2
Massage CAM on the body	132	21.3
Others (bathing, meditation)	28	4.5
Use of CAM with conventional medicine		
Use of CAM alone	340	56.3
Conventional medicine alone	271	43.7
Both CAM and conventional together	248	40.0

**Table 3:** Perceived benefit from CAM used.

Option	*f*	%
If benefited from CAM		
Yes	476	76.8
No	144	23.2
Examples of CAM benefited from		
Prayer	151	31.7
Herbs (for malaria, typhoid fever, and stomach upset)	189	39.7
Honey (for wound dressing, relief of cough)	208	43.7
Massage	26	5.5
Others (crude oil, python fat)	41	8.6
Perceived CAM effectiveness		
Effective with improved health status	453	73.1
Not effective	16	2.5
Cannot see any difference	151	24.4

**Table 4:** Perceived adverse effect of CAM used *n* = 620.

Option	*f*	%
Whether experienced adverse effect		
No	436	70.3
Yes	184	29.7
(a) Specific perceived adverse effects		
Cannot identify the actual adverse effect	116	63.0
Forever living product: generalized body discomfort	24	13.0
Herbs		
Dizziness	17	9.2
Weakness	15	8.1
Tuja 1000: diarrhea	6	3.3
Honey: stomach upset	6	3.3
Others: increase in body weight	5	2.7
Total	**184**	**100%**
